# Efficient induction of haploid plants in wheat by editing of *TaMTL* using an optimized *Agrobacterium*-mediated CRISPR system

**DOI:** 10.1093/jxb/erz529

**Published:** 2019-11-24

**Authors:** Huiyun Liu, Ke Wang, Zimiao Jia, Qiang Gong, Zhishan Lin, Lipu Du, Xinwu Pei, Xingguo Ye

**Affiliations:** 1 Institute of Crop Science, Chinese Academy of Agricultural Sciences, Beijing, China; 2 Biotechnology Research Institute, Chinese Academy of Agricultural Sciences, Beijing, China; 3 Shanghai Jiao Tong University, China

**Keywords:** *Agrobacterium*-mediated transformation, genome editing, haploid induction, *TaMTL*, *TaWaxy*, wheat

## Abstract

The use of CRISPR/LbCpf1 and CRISPR/xCas9 systems in wheat have not yet been reported. In this study, we compared the efficiencies of three CRISPR editing systems (SpCas9, LbCpf1, and xCas9), and three different promoters (*OsU6a*, *TaU3*, and *TaU6*) that drive single-guide (sg)RNA, which were introduced into wheat via *Agrobacterium*-mediated transformation. The results indicated that *TaU3* was a better choice than *OsU6a* or *TaU6*. The editing efficiency was higher using two sgRNAs than one sgRNA, and mutants with a large fragment deletion between the two sgRNAs were produced. The LbCpf1 and xCas9 systems could both be used successfully. Two endogenous genes, *TaWaxy* and *TaMTL*, were edited with high efficiency by the optimized SpCas9 system, with the highest efficiency (80.5%) being achieved when using *TaU3* and two sgRNAs to target *TaWaxy*. Rates of seed set in the *TaMTL*-edited T_0_ transgenic plants were much lower than that of the wild-type. A haploid induction rate of 18.9% was found in the *TaMTL*-edited T_1_ plants using the CRISPR/SpCas9 system. Mutants with reverse insertion of the deleted sequences of *TaMTL* and *TaWaxy* between the two sgRNAs were identified in the edited T_0_ plants. In addition, wheat grains lacking embryos or endosperms were observed in the *TaMTL*-edited T_1_ generation.

## Teaser

A comparison of different CRISPR systems and promoters using *Agrobacterium*-mediated transformation in wheat shows the SpCas9 system to be the most efficient for genome editing, and a high haploid induction rate is achieved by editing *TaMTL*.

## Introduction

Clustered regularly interspaced short palindromic repeats (CRISPR) is a type of bacterial defense system that degrades alien DNA, and it functions with various CRISPR-associated proteins (Cas9). Since its discovery, this system has been widely used in animals and plants for precise gene modification, especially the type-II CRISPR/SpCas9 system from *Streptococcus pyogenes*. The CRISPR/SpCas9 system is characterized by its efficiency and simplicity, and can recognize the protospacer-adjacent motif (PAM) site NGG with the assistance of trans-activating crRNA (tracrRNA). To date, genes of many crops have been edited using this technique, including maize, rice, wheat, soybean, barley, sorghum, potato, tomato, flax, and cotton (Ricroch *et al.*, 2017; Zhang *et al.*, 2018*a*; Abe *et al.*, 2019). The most frequent applications of the CRISPR/SpCas9 system are to produce gene-knockouts or null alleles, which are mainly achieved by the introduction of small indels that lead to frame-shift mutations. However, the CRISPR/SpCas9 system can only recognize DNA sequences upstream of the appropriate 5´-NGG-3´ PAMs, which limits the number of potential target sites. SpCas9 variants are therefore needed to overcome this restriction.

The recently identified type-II system, Cpf1 (CRISPR from *Prevotella* and *Francisella* 1), has distinct features compared to SpCas9. It is a single RNA-guided endonuclease that recognizes the thymidine-rich PAM and generates cohesive ends with four or five nucleotide overhangs rather than blunt-end breaks. Cpf1 is a dual nuclease that not only cleaves target DNA but also processes its own CRISPR RNA (crRNA) (Fonfara *et al.*, 2016; Zetsche *et al.*, 2017). Moreover, the maturation of crRNA by Cpf1 does not require the assistance of tracrRNA. The CRISPR/Cpf1 system also considerably expands the characteristics of SpCas9. It not only has genome-editing activity in mammalian cells, rice, Arabidopsis, soybean, and tobacco, but also has multiple gene-editing activity in mammalian cells and rice, where up to four genes can be simultaneously edited by Cpf1 using a single crRNA array spaced by mature direct repeats (Tang *et al.*, 2017; Wang *et al.*, 2017*b*; Zetsche *et al.*, 2017).

The SpCas9 variant xCas9, generated using phage-assisted continuous evolution, is reported to recognize a broad range of PAM sequences in mammals and rice, including NG, GAA, and GAT (Hu *et al.*, 2018; Wang *et al.*, 2018). The xCas9 3.7 system can recognize GAT, GAA, and NG PAM sites in mammalian cells and rice, and performs better than other Cas9 variants (Hu *et al.*, 2018; Wang *et al.*, 2018). Moreover, xCas9 can efficiently induce mutations at target sites with NG and GAT PAM sequences in rice. Hua *et al.* (2019) reported comparable editing efficiencies between xCas9 3.6 and xCas9 3.7 at the CGG, TGA, and CGT PAM sites, while xCas9 3.6 is 7.4 and 3.4 times as efficient as xCas9 3.7 at the AGC and GAT PAM sites, respectively. The most recent study found that xCas9 3.7 exhibits nearly equivalent editing efficiency to SpCas9 at most canonical NGG PAM sites, whereas it shows limited activity at non-canonical NGH (H=A, C, T) PAM sites (Zhong *et al.*, 2019*a*).

The editing efficiencies of CRISPR/Cas9 and CRISPR/Cpf1 have recently been compared in maize (Lee *et al.*, 2019), but a similar comparison has not yet been carried out in wheat. Although there have been some studies on the application of the CRISPR/SpCas9 system for genome editing in wheat (Wang *et al.*, 2014; Zhang et al., 2018*b*, 2019*b*), the use of the CRISPR/Cpf1 and CRISPR/xCas9 has not yet been reported. This may be due to the complicated genome of common wheat: many genes exist in at least three copies, and thus targeted genome editing is more difficult to achieve. In addition, wheat transformation is still a difficult task in many laboratories, and gene editing using CRISPR/Cpf1 and CRISPR/xCas9 requires a highly efficient transformation system as its basis.

Doubled-haploid technology substantially accelerates the breeding process for many crop species. In the past four decades, anther culture and microspore culture have been widely used to produce wheat haploid plants (Machii *et al.*, 1998; Liu *et al.*, 2002). However, strong genotype dependency exists in these two culture techniques as well as low plant regeneration frequency, and they involve complicated manipulation steps. Chromosome elimination techniques through wide crossing between wheat and maize or barley can generate wheat haploids, but well-controlled environmental are required conditions for growing the plants and for the rescue culture for the immature haploid embryos (Barclay 1975; Laurie *et al.*, 1986; Zhang *et al.*, 2014*b*). In maize, haploid plants can easily be obtained *in vivo* using an inbred haploid-inducer line, and many good inbred lines have been developed for efficient and fast breeding using this technique (Ishii *et al.*, 2016; Yao *et al.*, 2018).

Despite the problems, the CRISPR mutation system has great potential for editing economically important endogenous genes of wheat, such as *MATRILINEAL* (*MTL*) and *Waxy*. MTL is a pollen-specific phospholipase and can trigger haploid induction in maize by a frame-shift mutation (Kelliher *et al.*, 2017; Liu *et al.*, 2017). Knockout of *ZmDMP* can increase the haploid induction rate (HIR) by 5–6-fold in the presence of *MTL/ZmPLA/NLD* (Zhong *et al.*, 2019*b*). The knockout of *OsMATL* can reduce seed-set and lead to a 2–6% haploid induction rate in rice (Yao *et al.*, 2018). In wheat, three orthologs of *TaMTL*, namely *TraesCS4A02G018100*, *TraesCS4B02G286000*, and *TraesCS4D02G284700*, have been located on chromosomes 4A, 4B, and 4D, respectively. *Waxy* in wheat encodes granule-bound starch synthase I, which is required for the synthesis of amylose (Yamamori *et al.*, 1994) and influences starch composition and flour quality. In wheat, three orthologs of *TaWaxy*, namely *TraesCS4A02G418200*, *TraesCS7A02G070100*, and *TraesCS7D02G064300*, are located on chromosomes 4A, 7A, and 7D, respectively.

The objectives of this study were to develop a well-performing editing system for wheat, and to implement it to edit selected important example genes. First, we compared the efficiencies of the three editing systems, CRISPR/SpCas9, CRISPR/LbCpf1, and CRISPR/xCas9 using *Agrobacterium*-mediated transformation of a marker-free transgenic wheat line H29, which only carries a single copy of the *GUS* gene as the target (Wang *et al.*, 2017*a*; Liu *et al.*, 2020*b*). We then applied the optimized system to edit *TaMTL* and *TaWaxy* to develop the induction of haploids and to improve starch quality.

## Materials and methods

### Plant materials, plasmids, and bacteria strains

A marker-free transgenic wheat (*Triticum aestivum*) line H29 and the two varieties Fielder and Ningchun4 were used for transformation in this study. Line H29 was obtained by our laboratory from a transformation experiment using the cultivar Xinchun9 as a receptor, and it carries a single copy of the *GUS* gene and lacks the *bar* gene. In addition, *GUS* was inserted into the distal region of a pair of wheat chromosomes in H29 (Liu *et al.*, 2020*b*). Fielder and Ningchun4 were acquired from the Crop Germplasm Bank of China. All the plants were grown in pots (20×30 cm) in a growth chamber maintained at 24 °C, 16/8 h light/dark with 300 μmol m^–2^ s^–1^ light intensity at 45% humidity. For full details of cultivation see Wang *et al.*, (2017*a*).

The plasmid *pWMB110* containing the *bar* gene as a selection marker for generating transgenic plants and the maize (*Zea mays*) *ubi* promoter for driving the expression of a target gene or DNA sequence within a T-DNA region had previously been constructed by our laboratory (Supplementary Fig. S1 at *JXB* online). Vectors containing SpCas9 and LbCpf1 (*Lachnospiraceae bacterium* ND2006 Cpf1) were kindly provided by Prof. Yaoguang Liu (Southern China Agricultural University) and Prof. Chuanxiao Xie (Institute of Crop Sciences of Chinese Academy of Agricultural Sciences), respectively. *Agrobacterium* strain C58C1 and an *E. coli* strain containing the helper plasmid *pRK2013* were kindly provided by Dr Tom Clemente (University of Nebraska-Lincoln, USA).

### Construction of vectors for gene editing

The recombination vectors for the CRISPR/SpCas9, CRISPR/LbCpf1, and CRISPR/xCas9 systems were constructed based on methods described previously (Ma *et al.*, 2015; Wang *et al.*, 2017*b*; Hu *et al.*, 2018), and in addition xCas9 was synthesized using the sequence described by Hu *et al.* (2018). The promoters of *TaU3* (GenBank accession number X63065.1), *TaU6* (X63066.1), and rice (*Oryza sativa*) *OsU6a* (KR029106.1) were synthesized and cloned into the plasmid *pWMBX110-SpCas9* vector to generate the plasmids *pWMB110-SpCas9-TaU3*, *pWMB110-SpCas9-TaU6*, and *pWMB110-SpCas9-OsU6a* (Supplementary Fig. S1; see also Results). The *TaU3* promoter was also cloned into the vectors *pWMB110-LbCpf1* and *pWMB110-xCas9* to generate the plasmids *pWMB110-LbCpf1-TaU3* and *pWMB110-xCas9-TaU3*, respectively. The single-guide (sg)RNAs of the *GUS* gene (JN593326.1) were driven by the *TaU3*, *TaU6* and *OsU6a* promoters for the CRISPR/SpCas9 system and by the *TaU3* promoter for both the CRISPR/LbCpf1 and CRISPR/xCas9 systems. The sgRNAs of *TaMTL* and *TaWaxy* were all driven by the *TaU3* promoter. To target *GUS* in line H29, we designed both a single sgRNA (*g788*) and two sgRNAs (*g-22* and *g788*) for the CRISPR/SpCas9 system, two crRNAs (*g118* and *g2046*) for the CRISPR/LbCpf1 system, and three pairs of sgRNAs (*g788* and *g1718*, *g789* and *g1159*, *g1719* and *g1156*) for the CRISPR/xCas9 system (Supplementary Table S1). Two pairs of sgRNAs, one to target *TaMTL* (*TaMTL*-179 and *TaMTL*-471) and the other to target *TaWaxy* (*TaWaxy-296* and *TaWaxy-830*) were designed for the CRISPR/SpCas9 system.

The detailed single-guide (sg)RNA sequences of *GUS* were designed based on the respective PAM sites of the CRISPR/SpCas9, CRISPR/LbCpf1, and CRISPR/xCas9 systems and the restriction enzyme sites. The gRNA sequences of the three copies each of *TaMTL* and *TaWaxy* were designed by aligning their conserved sequences and further selecting 20 nucleotides upstream of a PAM motif (5´-NGG-3´) and restriction enzyme sites for the CRISPR/SpCas9 system (see Results). The primers for plasmid construction and detection of transgenic plants are listed in Supplementary Table S2. All the expression vectors were introduced into *Agrobacterium* strain C58C1 by triparental mating (Ditta *et al.*, 1980).

### *Agrobacterium*-mediated transformation

Immature wheat grains were collected 2 weeks after anthesis, sterilized with 75% ethanol for 1 min and 5% sodium hypochlorite for 15 min, and then washed five times with sterile water in aseptic conditions. Immature wheat embryos were isolated and *Agrobacterium*-mediated transformation was used to obtain transgenic plants following the protocol described by Wang *et al.* (2017*a*). Transgenic plants were transplanted into pots and cultivated in a growth chamber under the same conditions as described above.

### Detection analysis of edited mutations

Genomic DNA was extracted from candidate T_0_ transgenic mutant plants using a FastPure Plant DNA Isolation Mini Kit (Vazyme Biotech Co., Ltd) for digestion and deep sequencing. The targeted genes *GUS*, *TaMTL* and *TaWaxy* were amplified using their respective specific primers (Supplementary Table S2). A PCR-restriction enzyme (PCR-RE) assay was performed for these genes, where the reactions consisted of the corresponding restriction enzymes (1 U each) in 20 μl reaction buffer including 10 μl PCR product and were digested for 2 h at 37 °C. The resultant products were separated in a 2% agarose gel and visualized using a GelDoc XR System (BioRad). To distinguish different mutant types, the PCR products were subcloned into the pMD18-T vector (TaKaRa) and sequenced. For each mutant sample, at least five positive colonies were randomly selected and sequenced. The mutations were identified by aligning the reference sequences.

*GUS*-edited T_0_ transgenic plants that were detected by enzyme digestion or sequencing were confirmed by histochemical staining of fresh young leaves for GUS expression, as described by Jefferson *et al.* (1987).

### Cytological and phenotypic detection of TaMTL-edited mutations

Immature embryos at 2 weeks post-anthesis (from sterilized immature grains) of the *TaMTL*-edited T_0_ transgenic plants (as detected by enzyme digestion or Sanger sequencing) were cultured on MS medium. After 1 week of growth, the root tips from the germinated embryos were collected for chromosome observation as described by Han *et al.* (2004). The spikes and grains of the *TaMTL*-edited T_0_ transgenic plants at near-maturity stage were visually examined and rates for seed set were recorded.

Pieces of flag leaf of 2 cm length were sampled from the *TaMTL*-edited plants at the booting stage, and placed on glass slides with the abaxial epidermis uppermost. The mesophyll tissues of the samples were carefully scraped off using a sharp knife. The stomatal guard cells on the epidermis were observed and measured under an optical microscope incorporating a mirror micrometer (Zhang *et al.*, 2014*b*).

### Quantitative reverse-transcriptase PCR

Anther was sampled from the *TaMTL*-edited plants QD33-3, QD33-14, and QD33-26 and the wild-type Fielder plants in order to determine the transcript abundances of *TaMTL-4A*, *TaMTL-4B*, and *TaMTL-4D* by quantitative reverse-transcriptase (qRT-)PCR. Total RNA was extracted using Trizol reagent (Invitrogen). cDNA synthesis was then carried out using HiScript III RT SuperMix for qPCR (+gDNA wiper) (Vazyme Biotech Co., Ltd), and 1 μg of the total RNA was reverse-transcribed using 5×HiScript III qRT SuperMix at 37 °C for 15 min and at 85 °C for 5 s. Finally, qRT-PCR was performed using ChamQ Universal SYBR qPCR Master Mix (Vazyme Biotech Co., Ltd) in a 7500 Fast Real-Time PCR system (Applied Biosystems). The specific primers for *TaMTL-4A*, *TaMTL-4B*, and *TaMTL-4D* are listed in Supplementary Table S2. Transcript abundance was expressed relative to that of *TaADP* using the 2^–2ΔΔ*C*T^ method (Livak and Scmittgen, 2001).

## Results

### Optimization of promoters for sgRNA regulation in the CRISPR/SpCas9 system

**Fig. 1.**
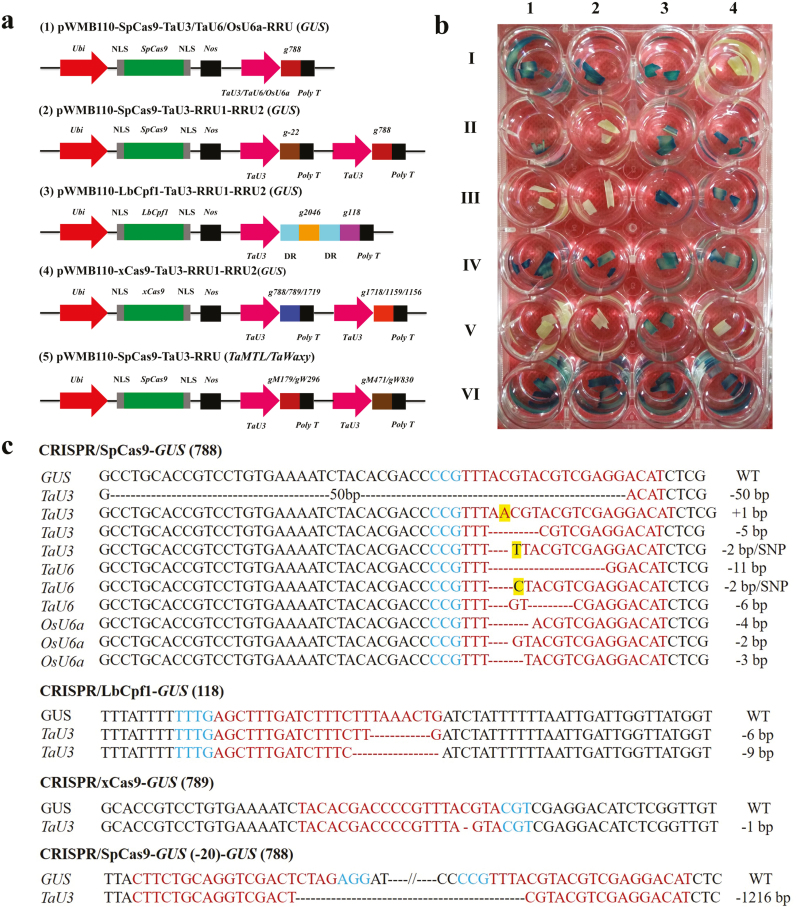
Genome editing in wheat using the CRISPR/SpCas9, CRISPR/LbCpf1, and CRISPR/xCas9 systems. (a) Linearized CRISPR/SpCas9, CRISPR/LbCpf1, and CRISPR/xCas9 constructs. *ubi*, *Zea mays ubi* promoter; NLS, nuclear localization signal; *Nos*, *Nos* terminator; *TaU3*/*TaU6*/*OsU6a*, different sgRNA promoters; *Poly T*, a 7-bp poly T sequence; RRU, sgRNA (*GUS*, *TaWaxy*, *TaMTL*) or crRNA (*GUS*) ribozyme units; DR, direct repeat. (b) The expression of GUS protein in edited T_0_ transgenic plants. The heterozygous mutants are I1–2, II3–4, III3–4, IV1–4; the bi-allelic mutants are II2, III1–2, V1–2, 4; non-mutant plants are V3, VI1–4., VI2, VI3 and VI4; H29 is I3; and wild-type Xinchun9 is I4. (c) InDel mutations in *GUS* from edited T_0_ transgenic plants. Blue letters indicate the PAM sequences, red letters indicate the sgRNA or crRNA sequences, and the dashed lines represent nucleotide deletions. Insertions and SNPs are shaded in yellow, and the size of the deletion or insertion is shown on the right. SNP, single-nucleotide polymorphism.

Editing efficiencies were determined using three promoters for controlling sgRNA expression: the rice *U6* promoter (*OsU6a*), and the wheat *U3* (*TaU3*) and *U6* promoters (*TaU6*). The widely used SpCas9 was driven by the maize ubiquitin promoter, and the same sgRNA targeting *GUS* was controlled by the *OsU6a*, *TaU3*, or *TaU6* promoters (Fig. 1a). The three different vectors were transformed using *A. tumefaciens* into the marker-free transgenic wheat line H29, which only has a single copy of *GUS*. PCR-RE assays and Sanger sequencing showed that the editing efficiencies of the different promoters in plants of the T_0_ generation were very different (Table 1, Supplementary Fig. S2). The editing efficiency of the *OsU6a* promoter was only 21.6% and most of the mutant plants were heterozygous. The editing efficiency of the *TaU6* promoter was 36.0%, and only 4.9% of the mutants were bi-allelic (3 out of 61 plants) while 31.1% were heterozygous (19 out of 61 plants). Interestingly, the mutation efficiency was 61.4% for the *TaU3* promoter; moreover, 20.0% and 41.4% of the mutants were bi-allelic and heterozygous, respectively (Table 1). These results clearly demonstrated that of the three promoters tested, *TaU3* was the best choice for driving sgRNA expression in *Agrobacterium*-mediated genome editing in wheat.

**Table 1.** Summary of the target sequences and mutations in wheat T_0_ plants obtained using the CRISPR/SpCas9, /LbCpf1, and /xCas9 editing systems

Target loci	Promoter	PAM-guide sequence (5´–3´)	No. of transgenic plants	No. of mutant plants	Mutation rate %	Genotypes obtained
**SpCas9-S (one sgRNA)**						
*GUS*-788	*TaU3*	**CCG**TTTACGTACGTCGAGGACAT	70	43	61.4	14Bi + 29He + 27WT
*GUS*-788	*TaU6*	**CCG**TTTACGTACGTCGAGGACAT	61	22	36.0	3Bi + 19He + 39WT
*GUS*-788	*OsU6a*	**CCG**TTTACGTACGTCGAGGACAT	74	16	21.6	2Bi + 14He + 58WT
**SpCas9-D (two sgRNAs)**						
*GUS*--22	*TaU3*	CTTCTGCAGGTCGACTCTAG**AGG**	164	75	45.7	24Bi + 51He + 89WT
*GUS*-788	*TaU3*	**CCG**TTTACGTACGTCGAGGACAT	164	106	64.6	34Bi + 72He + 58WT
**LbCpf1**						
*GUS*-118	*TaU3*	**TTTG**AGCTTTGATCTTTCTTTAAACTG	65	2	3.1	2He + 63WT
*GUS*-2046	*TaU3*	**TTTC**GGCTACAAGAACGCTAGCCATCAC	65	0	0	65WT
**xCas9 3.7**						
*GUS*-788	*TaU3*	**CCG**TTTACGTACGTCGAGGACAT	71	32	45.1	32He + 39WT
*GUS*-789	*TaU3*	TACACGACCCCGTTTACGTA**CGT**	67	1	1.5	1He + 66WT

PAM, protospacer-adjacent motif, with the target sequences highlighted in bold. SpCas9-S, containing a single sgRNA; SpCas9-D, containing two sgRNAs; Bi, bi-allele; He, heterozygote; WT, wild-type.

### Comparison of the editing efficiencies of the three CRISPR systems

Two sgRNAs (*g-22* and *g788*) together targeting *GUS* were designed to be driven by the *TaU3* promoter and their total editing efficiencies were compared with the single sgRNA *g788* targeting the *GUS* gene (Fig. 1a). Among the 164 transgenic plants obtained, PCR-RE assays and Sanger sequencing identified 75 and 106 plants with mutations at the *g-22* and *g788* sites, respectively, and 70 plants with simultaneous mutations at both sites. Their editing efficiencies were 45.7%, 64.6%, and 42.7%, respectively (Table 1, Supplementary Table S3). The total editing efficiency was up to 70.1% for plants in which a mutation occurred in one or two loci (Supplementary Table S3). Simultaneous mutations at the two sites, which can lead to a large fragment deletion, had an efficiency of 37.2% (Table 2). The results indicated that the combination of the two sgRNAs could result in a higher editing efficiency compared to a single sgRNA. Furthermore, the combination of sgRNAs could lead to a large fragment deletion.

**Table 2.** Summary of the target sequences and mutations of *GUS*, and different *TaMTL* and *TaWaxy* homologous genes in T_0_ wheat plants edited using the CRISPR/SpCas9-D editing system containing two sgRNAs

Target loci	PAM-guide sequence (5´–3´)	No. of transgenic plants	No. of mutant plants	Mutation rate %	Genotypes obtained	LFD	LFD rate (%)
*GUS*-22	CTTCTGCAGGTCGACTCTAG**AGG**	164	75	45.7	24Bi + 51He + 89WT	61	37.2
*GUS*-788	**CCG**TTTACGTACGTCGAGGACAT		106	64.6	34Bi + 72He + 58WT		
*TaMTL4A*-179	**CCA**AGCTGCAGGAGCTGGACGGC	101	35	34.7	11Bi + 24He + 66WT	9	8.9
*TaMTL4A*-471	**CCG**CGGTGACCGCATCGCTGAGG		36	35.6	13Bi + 23He + 65WT		
*TaMTL4B*-179	**CCA**AGCTGCAGGAGCTGGACGGG	101	19	18.8	6Bi + 13He + 82WT	6	5.9
*TaMTL4B*-471	**CCG**CGGTGACCGCGTCGCTGAGG		9	8.9	7Bi + 2He + 92WT		
*TaMTL4D*-179	**CCA**AGCTGCAGGAGCTGGACGGG	101	24	23.8	13Bi + 11He + 77WT	8	7.9
*TaMTL4D*-471	**CCG**CGGTGACCGCGTCGCTGAGG		16	15.8	8Bi + 8He + 85WT		
*TaWaxy4A*-296	GGCGGCCTCGGCGACGTCCT**CGG**	87	35	40.2	25Bi + 10He + 52WT	10	11.5
*TaWaxy4A*-830	AAGACCAAGGAGAAGATCTA**CGG**		36	41.4	29Bi + 7He + 51WT		
*TaWaxy7A*-296	GGCGGCCTCGGCGACGTCCT**CGG**	87	39	44.8	19Bi + 20Bi + 48WT	11	12.6
*TaWaxy7A*-830	AAGACCAAGGAGAAGATCTA**TGG**		47	54.0	25Bi + 22He + 40WT		
*TaWaxy7D*-296	GGCGGCCTCGGCGACGTCCT**CGG**	87	23	26.4	10Bi + 13He + 64WT	14	16.1
*TaWaxy7D*-830	AAGACCAAGGAGAAGATCTA**CGG**		31	35.6	18Bi + 13He + 56WT		

PAM, protospacer-adjacent motif, with the target sequences highlighted in bold. Bi, bi-allele; He, heterozygote; WT, wild-type; LFD, large fragment deletion.

The *TaU3* promoters were also used to drive the expression of two sgRNAs targeting *GUS* in order to compare the editing efficiencies of CRISPR/SpCas9, CRISPR/xCas9, and CRISPR/LbCpf1 in wheat. The PAM site for LbCpf1 is TTTN, and two sgRNAs (*g118* and *g2046*) with direct repeats (DRs) were designed to target this site (Fig. 1a). PCR-RE assays and Sanger sequencing identified only two of the 65 transgenic plants as having mutations in the *g118* target, and the editing efficiency was only 3.1% (Table 1). The two plants were heterozygous mutants; one had a 6-bp deletion and the other had a 9-bp deletion in the target sequence (Fig. 1c). In contrast, no mutations were detected within the *g2046* target (Table 1).

The xCas9 3.7 system was employed to edit *GUS* at different PAMs, namely NGG (788), NGA (1718), NGT (789), NGC (1159), GAA (1719), and GAT (1156), and the target sequences of NGG and NGA, NGT and NGC, and GAA and GAT were designed as pairs in the CRISPR/xCas9 vector (Fig. 1a). Out of 71 transgenic plants obtained, PCR-RE assays and Sanger sequencing identified 32 plants with mutations at the NGG PAM site, with an editing efficiency of 45.1%. All the mutant plants were heterozygous. With regards to the NGT PAM site, only one mutant was identified out of 67 transgenic plants obtained (Table 1); this plant was heterozygous with a 1-bp deletion (Fig. 1c). There were no mutations detected at the NGA, NGC, GAA, or GAT PAM sites in the 71, 67, 79, and 79 transgenic plants obtained, respectively. Even though the target sequence was the same in the two systems xCas9 edited the NGG PAM site with lower efficiency than SpCas9.

### Inheritance of the mutation sites in the edited plants

The segregation of the bi-allelic and heterozygous mutants derived from the CRISPR/SpCas9 system were examined in the T_1_ generation, in which the expression of the sgRNA for *GUS* was driven by the *TaU3* promoter. All the original bi-allelic mutants were homozygous or bi-allelic in the T_1_ generation and 21 transgene-free mutants were obtained from a total of 87 plants. Of the T_1_ progeny from the mutants that were heterozygous in the T_0_ generation, 19 were homozygous, 56 were heterozygous, and 20 had no mutations, as determined by PCR-RE assays. The T_1_ segregation ratio was 1:2:1 (*P*>0.05) and in accordance with Mendelian heritance patterns. *GUS* was silenced in the homozygous and bi-allelic mutants in which the deleted base pairs could not be divided by 3 (Fig. 1b), implying that the *GUS* gene-silencing mutations were induced by the editing system. Sanger sequencing revealed that all the mutations in *GUS* in the T_1_ generation were consistent with those in the T_0_ generation and no additional mutations were observed. These results demonstrated that CRISPR/SpCas9 induced-mutations were heritable in wheat.

### Confirmation of the optimized CRISPR system by editing the endogenous genes *TaMTL* and *TaWaxy*

**Fig. 2.**
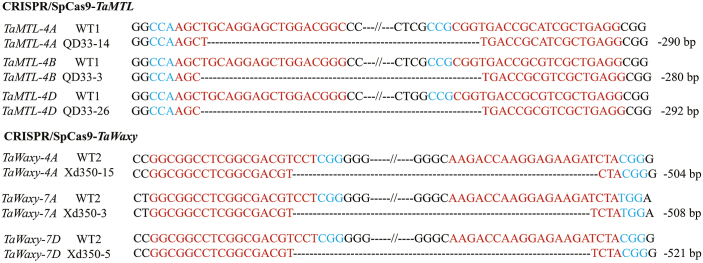
Sequences of the large fragment deletions in *TaMTL* and *TaWaxy* in different loci of chromosomes of wheat plants edited using the CRISPR/SpCas9 system. WT1, Fielder variety (wild-type); WT2, Ningchun4 variety (wild-type). *TaMTL*-edited plants are named as QD33, and *TaWaxy*-edited plants are named as Xd350. Blue letters indicate the PAM sequences, red letters indicate the sgRNA sequences, and the dashed lines represent nucleotide deletions.

**Fig. 3.**
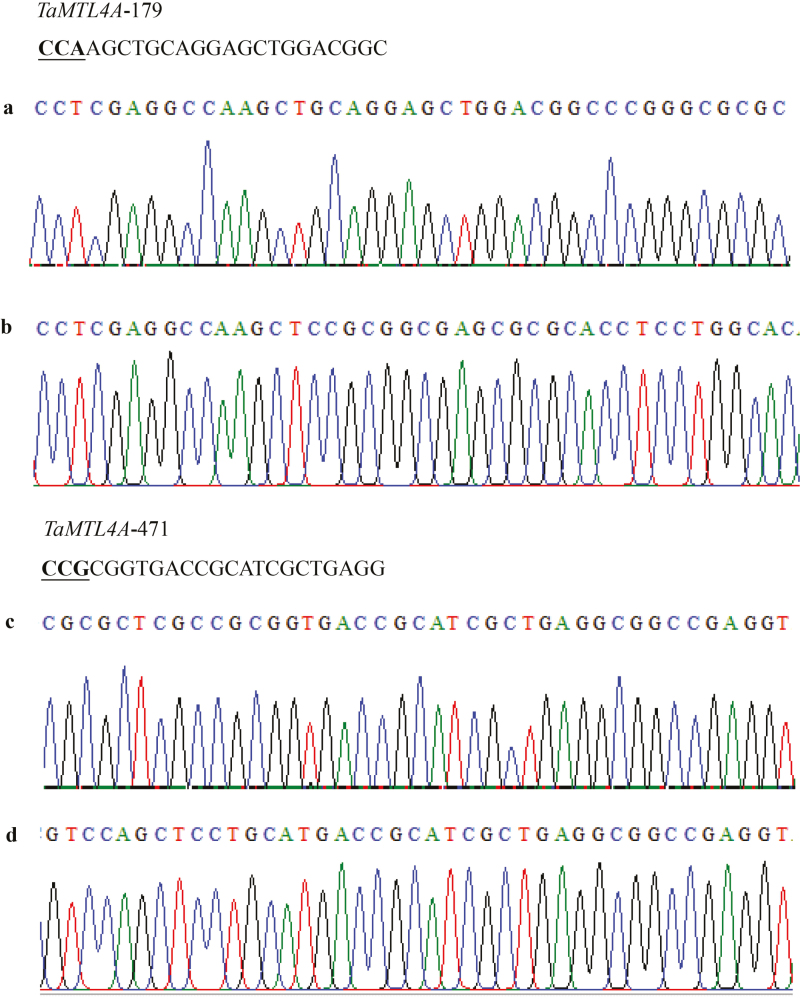
Detailed reverse sequence insertion analysis in wheat *TaMTL* at the 4A chromosomal loci. (a, c) *TaMTL*-4A sequence in the wild-type Fielder variety, and (b, d) *TaMTL*-4A sequence in QD33-3, which was edited using the CRISPR/SpCas9 system.

**Fig. 4.**
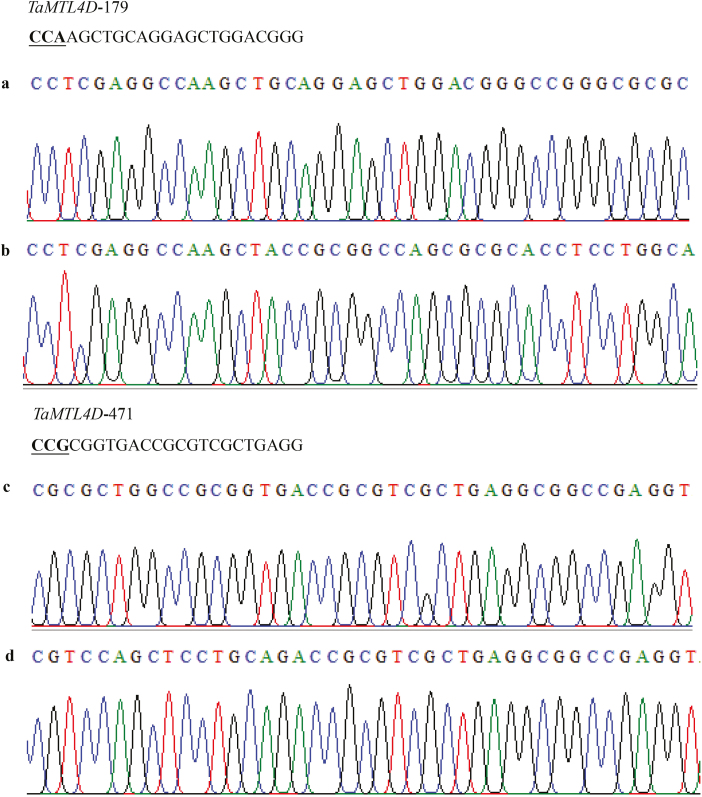
Detailed reverse sequence insertion analysis in wheat *TaMTL* at the 4D chromosomal loci. (a, c) *TaMTL*-4D sequence in the wild-type Fielder variety, and (b, d) *TaMTL*-4D sequence in QD33-3, which was edited using the CRISPR/SpCas9 system.

The CRISPR/SpCas9 system was further used to edit two wheat endogenous genes, *TaMTL* and *TaWaxy*. The expression of the sgRNAs of the two genes was driven by the *TaU3* promoter, and the constructs were introduced into the varieties Fielder and Ningchun4 via *Agrobacterium*-mediated transformation (Fig. 1a). For *TaMTL*, two sgRNAs (*TaMTL*-179 and *TaMTL*-471) were designed to target the first and second exons of the three homologous genes on chromosomes 4A, 4B, and 4D genomes in Fielder. The editing efficiencies of this gene were up to 46.5% and 38.6% at the targets of *TaMTL-179* and *TaMTL-471*, respectively (Table 2, Supplementary Fig. S2). The total editing efficiency was up to 57.5% for plants in which a mutation occurred at one or two loci (Table 3). At the target *TaMTL-179*, the editing efficiencies for the homologous genes on chromosomes 4A, 4B, and 4D were 34.7%, 18.8%, and 23.8%, respectively (Table 2; Supplementary Figs S2, S3), and at the target *TaMTL-471*, the efficiencies for the homologous genes on the three chromosomes were 35.6%, 8.9%, and 15.8%, respectively (Table 2; Supplementary Figs S2, S3). The editing efficiency was 12.9% for simultaneous mutations at the three loci and 13.9% for simultaneous mutations at two loci (Table 3). In addition, a large fragment deletion was also identified when the two targets were simultaneously edited on any one of chromosomes 4A, 4B, or 4D, with efficiencies of 8.9%, 5.9%, and 7.9% for the three homologous genes, respectively (Fig. 2, Supplementary Fig. S5a, Table 2). Interestingly, we found a mutant, QD33-3, with reverse insertion of the deleted sequences within the *TaMTL* homologous genes on chromosomes 4A and 4D in the T_0_ generation, which lays a foundation for target gene replacement in wheat (Fig 3, Fig. 4, Supplementary Figs S6, S7).

**Table 3.** Frequency distribution of the chromosomal locus mutations for *TaMTL* and *TaWaxy* in T_0_ wheat plants using the CRISPR/SpCas9 system

	Number of loci			
Mutation	3	2	1	0
*TaMTL*	13 (12.9%)	14 (13.9%)	31 (30.7%)	43 (42.5%)
*TaWaxy*	28 (32.2%)	20 (23.0%)	22 (25.3%)	17 (19.5%)

**Fig. 5.**
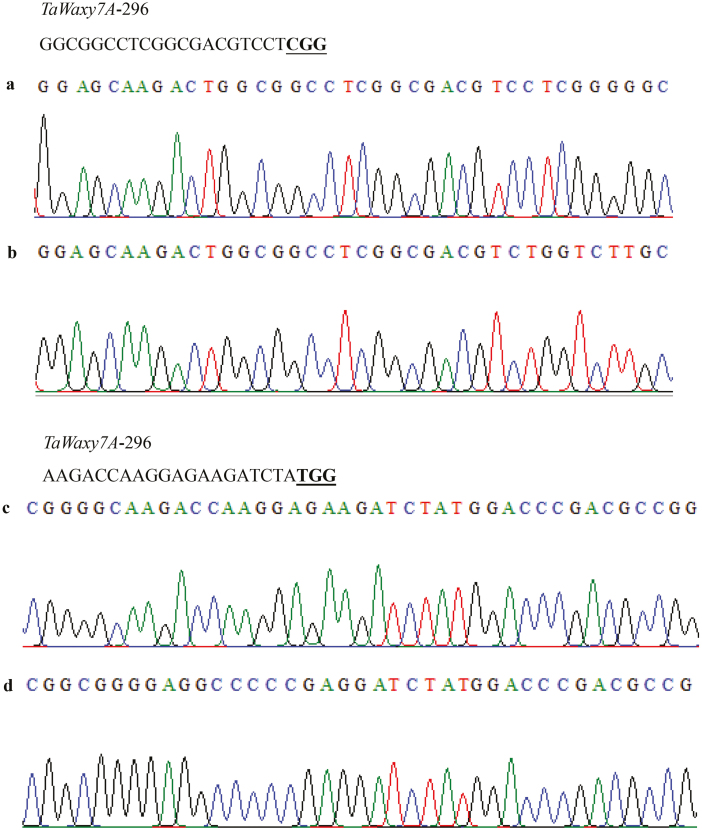
Detailed reverse sequence insertion analysis in wheat *TaWaxy* at the 7A chromosomal loci. (a, c) *TaWaxy*-7A sequence in the wild-type Ningchun4 variety, and (b, d) *TaWaxy*-7A sequence in Xd350-15, which was edited using the CRISPR/SpCas9 system.

The editing efficiencies of the sgRNAs targeting *TaWaxy-296* and *TaWaxy-830* on the first and fourth exons of chromosomes 4A, 7A, and 7D in the widely cultivated commercial hexaploid variety Ningchun4 were as high as 47.1% and 71.3%, respectively (Table 2; Supplementary Fig. S2). The total editing efficiency was up to 80.5% for mutations occurring at one or two loci (Table 3). At the target *TaWaxy-296*, the editing efficiencies for the three homologous genes on chromosomes 4A, 7A, and 7D were 40.2%, 44.8%, and 26.4%, respectively (Table 2, Supplementary Fig. S8), and at the target *TaWaxy-830*, the efficiencies were 41.4%, 54.0%, and 35.6%, respectively (Table 2, Supplementary Fig. S9). The efficiency was 32.2% for simultaneous mutations at the three loci and 23.0% for simultaneous mutations at two loci (Table 3). In addition, a large fragment deletion was also identified where the two targets were simultaneously edited on chromosomes 4A, 7A, and 7D, and the efficiencies were 11.5%, 12.6%, and 16.1%, respectively (Fig. 2, Supplementary Fig. S5b, Table 2). We also identified a mutant, Xd350-15, with reverse insertion of the deleted sequences within the *TaWaxy* homologous genes on chromosome 7A in the T_0_ generation (Fig. 5, Supplementary Fig. S10). Four selected *TaWaxy*-edited lines (Xd350-3, Xd350-5, Xd350-8, and Xd350-15) were found to be simultaneous mutations in the three homologous genes *TaWaxy-4A*, *TaWaxy-7A*, and *TaWaxy-7D* (Fig. 2, Supplementary Figs S8, S9).

### Detection of off-target mutations using Sanger sequencing

We next assessed the potential off-target effects using *GUS*, *TaMTL*, and *TaWaxy* in the CRISPR/SpCas9, CRISPR/LbCpf1, and CRISPR/xCas9 systems. The potential off-targets of the homologous positions of the sgRNAs for these genes were searched in the wheat reference genome (EnsemblPlants: http://plants.ensembl.org/Triticum_aestivum/Tools/Blast), and six potential off-target sites were found for *GUS*, six for *TaMTL*, and four for *TaWaxy*. Compared with the target sequences they had 2–4 bp mismatches (Supplementary Table S4). To detect the off-target events, we designed specific primers and amplified possible off-target areas in the T_0_ transgenic plants that contained the guide RNA (Supplementary Table S4) and then the PCR amplicons were detected by Sanger sequencing. No mutation was found to occur at all the 16 possible off-target sites. These results indicated that the possible off-targets could be ignored, and that the CRISPR system could highly specifically target the selected sites for mutation in the edited plants obtained in this study.

### Editing of *TaMTL* induces a reduction in seed set and an increase in haploid production

**Fig. 6.**
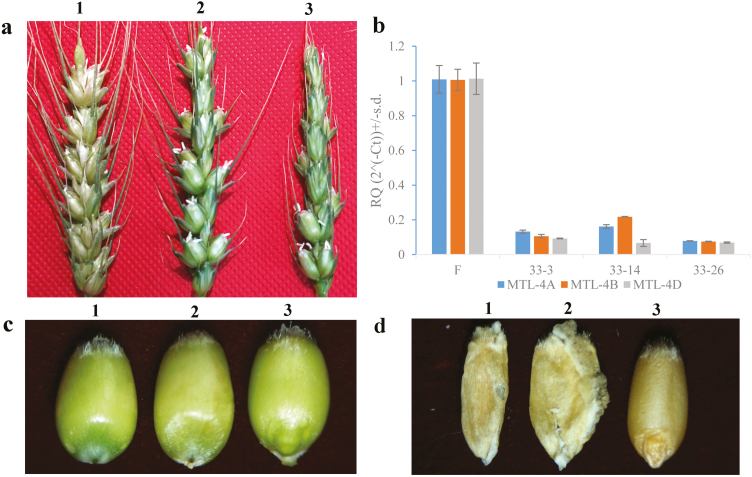
Phenotypes of *TaMTL*-edited wheat lines using the CRISPR/SpCas9 system. (a) Spikes of the wild-type Fielder variety (1) and the *TaMTL*-knockout plants (2, 3). (b) Relative amounts of *TaMTL-4A*, *TaMTL-4B*, and *TaMTL-4D* RNA in wild-type Fielder (F) and *TaMTL*-edited plants (QD33-3, QD33-14, and QD33-26). The expression of the wild-type was set to 1 for each gene. (c) Seeds of *TaMTL*-edited plants with no embryo (1, 2) compared with the wild-type (3). (d) Seeds of *TaMTL*-edited plants with no endosperm (1, 2) compared with the wild-type (3).

Of the 55 *TaMTL*-edited plants, seven lines were examined further: QD33-3, QD33-14, QD33-20, QD33-26, QD33-43, QD33-46, and QD33-52 (Figs 2–4, Supplementary Figs S6, S7 at *JXB* online). There was a large fragment deletion (290 bp) between the two targets in the homologous genes on chromosome 4A in QD33-14, QD33-20, and QD33-26. Lines QD33-3 and QD33-43 had a large fragment deletion (280 bp) on chromosome 4B, and there was a reverse insertion between the two targets in the homologous genes on chromosomes 4A and 4D in QD33-3. Another large fragment deletion (292 bp) was found in QD33-26 and QD33-46 between the two targets in the homologous genes on chromosome 4D. Seed-set rates of the *TaMTL*-edited T_0_ transgenic plants were much lower than that of the wild-type Fielder (Fig. 6a, Table 4). Among them, *TaMTL-4A*, *TaMTL-4B*, and *TaMTL-4D* were simultaneously edited in QD33-3, QD33-14, QD33-20, QD33-26, QD33-43, and QD33-46, and *TaMTL-4A* and *TaMTL-4D* were simultaneously edited in QD33-52 (Table 4). qPCR analysis showed that the post-transcriptional expression levels of *TaMTL-4A*, *TaMTL-4B*, and *TaMTL-4D* in the anthers of plants QD33-3, QD33-14, and QD33-26 were significantly reduced in comparison with the wild-type (Fig. 6b).

**Table 4.** Seed-set and haploid induction rates of *TaMTL*-edited plants of wheat using the CRISPR/SpCas9 system

Plant ID	Genotype of *TaMTL-4A*	Genotype of *TaMTL-4B*	Genotype of *TaMTL-4D*	TSSN	RSSN	No. of seeds with no embryo	SSR (%)	No. of haploid plants	HIR (%)
QD33-3	Bi	L	Bi	86	19	2	22.1	3	26.3
QD33-14	L	He	He	86	17	2	19.8	0	11.8
QD33-20	L	He	Bi	89	21	3	23.6	1	19.0
QD33-26	L	Bi	L	85	19	2	22.4	4	31.6
QD33-43	Bi	L	Bi	84	17	0	20.2	2	11.8
QD33-46	He	He	L	87	19	1	21.8	3	21.1
QD33-52	Bi	WT	Bi	90	20	1	22.2	1	10.0
Fielder	WT	WT	WT	92	83	0	90.2	0	0

Bi, bi-allele; He, heterozygote; L, large fragment deletion; WT, wild-type; TSSN, theoretical seed-set number; RSSN, real seed-set number; SSR, seed-set rate; HIR, haploid induction rate.

**Fig. 7.**
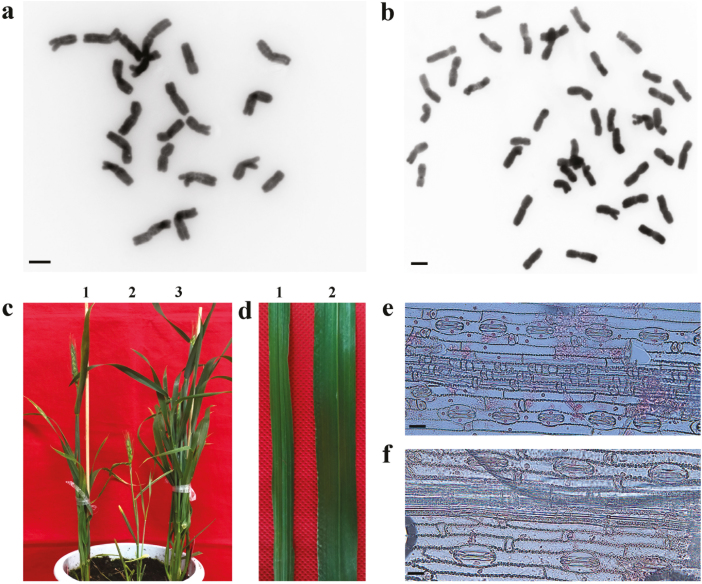
Characteristics of the wheat haploid plants in the *TaMTL*-edited mutants using the CRISPR/SpCas9 system. (a, b) Chromosomes of haploids (a) and diploids (b) in *TaMTL*-edited T_1_ plants. The haploid plants had 21 chromosomes and the diploid plants had 42. (c) Plant height in the haploid (2) and the diploid (1, 3). (d) Leaf width in the haploid (1) and the diploid (2). Guard cell length in the haploid (e) and the diploid (f). Scale bars in (a, b, e, f) are 10 μm.

Interestingly, some grains lacking an embryo were found in QD33-3, QD33-14, QD33-20, QD33-26, QD33-46, and QD33-52 (Fig. 6c), and two shriveled grains without endosperm and embryo were found in QD33-3 (Fig. 6d). The ploidy of the immature seeds in the *TaMTL*-edited plants was determined through chromosome counting, and 25 haploids were identified from a total of 132 seeds: the haploid seeds had 21 chromosomes while the diploid seeds had 42. The haploids had shorter plant height, fewer tillers, narrower leaves, and shorter guard-cell length compared with the diploids (Fig. 7). A haploid induction rate of 10.0–31.6% was observed in the gene-edited plants (Table 4).

## Discussion

The CRISPR/Cas9 system has been widely used for plant genome editing since its first development and, as an example, a number of wheat genes have been edited with this system over the last year by *Agrobacterium*-mediated transformation (Abe *et al.*, 2019; Liu *et al.*, 2020*a*; Okada *et al.*, 2019; Zhang *et al.*, 2019*b*). However, the editing efficiency is lower in wheat than in other plants (Ma *et al.*, 2015; Feng *et al.*, 2018). In the genome editing of other plant species including maize and rice, the promoters used for both the sgRNAs and *Cas9* are thought to significantly influence the targeting efficiency (Xu et al., 2014; Zhang *et al.*, 2014*a*; Ma *et al.*, 2015; Xie *et al.*, 2015; Zhu *et al.*, 2016; Feng *et al.*, 2018). The *ubi* promoter works better than the *CaMV35S* promoter for regulating *Cas9* in rice, Arabidopsis, and maize (Feng *et al*, 2014; Xing *et al.*, 2014). The sgRNA sites are also very important for the generation of target mutations in the gene of interest and greatly influence the gene-editing efficiency (Zhu *et al.*, 2016; Feng *et al.*, 2018). In the present study, the maize *ubi* promoter was used to control *SpCas9*, and the wheat *TaU3* and *TaU6* and rice *OsU6a* promoters were used to control *sgRNAs* to edit *GUS* by *Agrobacterium*-mediated transformation. The editing efficiencies of *TaU3*, *TaU6*, and *OsU6a* were 61.4%, 36.0%, and 21.6%, respectively (Table 1). The *TaU3* promoter thus appeared to be the best choice for regulating sgRNA expression when performing *Agrobacterium*-mediated CRISPR gene editing in wheat. When two sgRNAs were designed to target *GUS*, the editing efficiency was up to 70.1% (Supplementary Table S3), and large fragment deletions were also detected between the two targets with efficiencies up to 37.2% (Table 2). The editing efficiency using two sgRNAs was therefore higher than by using a single sgRNA to target the gene. Interestingly, although editing mutations including large fragment deletions were detected in *TaMTL*-edited, *TaWaxy*-edited, and *GUS*-edited plants, the efficiencies for *TaMTL-4A*, *TaMTL-4B*, and *TaMTL-4D*, and *TaWaxy-4A*, *TaWaxy-7A*, and *TaWaxy-7D* were lower than that for *GUS* (Table 2). *GUS* was an exogenous gene that was inserted onto the distal region in a pair of wheat chromosomes with a single copy in the H29 line used in this study (Liu *et al.*, 2020*b*), while the endogenous *TaMTL* and *TaWaxy* genes were all present in the wheat genome with three copies. We therefore conclude that the copy number and chromosomal locations of the target genes might influence the editing efficiencies.

Compared with CRISPR/SpCas9, CRISPR/xCas9 edited the NGG PAM site with lower efficiency even though the target sequence was the same in the two systems, which was consistent with a previous report (Wang *et al.*, 2018). In the xCas9 system, the target sequence in the NGT PAM site was almost similar to the NGG PAM site, but its editing efficiency was quite low. In addition, although the target sequences at the NGA, NGC, GAA, and GAT PAM sites in xCas9 were different from the sequence at the NGG PAM site in the SpCas9 system, no mutant plant was detected at all four sites. The editing efficiencies at the two target sites in CRISPR/LbCpf1 (*g118* and *g2046*) and the six target sites in xCas9 (*g788*, *g1718*, *g789*, *g1159*, *g1719*, and *g1156*) were clearly much lower than those at the two target sites in SpCas9 (*g-22* and *g788*) (Table 1, Supplementary Table S1). A recent study found that xCas9 possesses limited activity at non-canonical NGH (H=A, C, T) PAM sites in rice protoplasts (Zhong *et al.*, 2019*a*), and other studies have also reported that the editing efficiency of Cpf1 in maize and Arabidopsis is significantly lower than that in rice (Tang *et al.*, 2017; Wang *et al.*, 2017*b*; Lee *et al.*, 2019; Malzahn *et al.*, 2019). We therefore conclude that species diversity may be the reason for the lower editing efficiencies of the CRISPR/LbCpf1 and CRISPR/xCas9 systems in wheat than in other plants.

The genetic behavior of the edited *GUS* gene indicated that its segregation was accordance with Mendelian heritance patterns, and all the mutations of *GUS* in the T_1_ generation were consistent with those in the T_0_ generation with no additional mutations being observed. This was consistent with the report by Howells *et al.* (2018) in which no additional editing was observed in the T_1_ generation. In a recently published study, it was found that some new mutations happened and editing efficiencies increased in the next a few generations (Zhang *et al.*, 2019*a*). Our results were different because we selected edited plants for detailed investigation at target sites that might not have harbored integration of SpCas9 and sgRNA. Generally, the transgenic plants might have been chimeric in T_0_, and some new edited plants might have missed detection in this generation but gone on to be detected in the next generation. Moreover, wheat genome editing might be closely associated with the design of target sites, application of promoters for sgRNA, and location of target genes (Howells *et al.*, 2018).

By combining gene-editing technology with haploid induction technology important genes can be edited, and in addition homozygous edited plants can be quickly obtained. For example, Kelliher *et al.* (2019) transformed a vector expressing *Cas9* and a gRNA targeting the putative wheat *GRASSY TILLER1* orthologs *TaGT1-4A*, *TaGT1-4B*, and *TaGT1-4D* into a maize inbred line NP2222 and then this was pollenated to wheat, resulting in haploid and doubled-haploid wheat plants with the edited *GRASSY TILLER1*. The key gene controlling the haploid production trait in maize, *ZmPLA*, has recently been cloned, and knockout of this gene by CRISPR/Cas9 leads to the production of haploid seeds at a rate of 6–10% (Dong *et al.*, 2018). Editing the homologous gene of *ZmPLA* to silence it in rice by using CRISPR/Cas9 resulted in the formation of haploid grains at a rate of 2–6% (Yao *et al.*, 2018). In our study, we used an optimized CRISPR/SpCas9 system and edited wheat *TaMTL*, which is the homolog of *ZmPLA* for haploid induction. Our results showed that a double-knockout mutation of *TaMTL-4A* and *TaMTL-4D* resulted in haploid induction at a frequency of 10%, and triple-knockout of *TaMTL-4A*, *TaMTL-4B*, and *TaMTL-4D* resulted in haploid induction at a frequency of 11.8–31.6% (Table 4). A new study has recently reported that *TaPLA-A* and *TaPLA-D* knockout lines trigger haploid induction at a rate of 2–3% (Liu *et al.*, 2020*a*). In our CRISPR/SpCas9 system, we used the *TaU3* promoter to control two sgRNAs to target *TaMTL-4A*, *TaMTL-4B*, and *TaMTL-4D* simultaneously, whereas Liu *et al.* (2020*a*) used the *TaU6* promoter to regulate one sgRNA to target *TaPLA*. Clearly, our haploid induction rate was the highest. In addition, we not only identified haploid plants in the T_1_ generation from the lines QD33-3, QD33-14, QD33-20, QD33-26, QD33-43, QD33-46, and QD33-52, but also found some grains lacking an embryo in the lines QD33-3, QD33-14, QD33-20, QD33-26, QD33-46, and QD33-52. Interestingly, two shriveled grains without endosperm and embryo were found in the line QD33-3 (Fig. 6c, d). Further research is needed to determine the mechanism of endosperm and embryo abortion.

The starch composition of wheat grains has an important influence on flour quality. Amylose is encoded by the *Waxy* gene, which is synthesized by granule-bound starch synthase I (Sano, 1984; Wang *et al.*, 1990). In common wheat, *Waxy* is located on chromosomes 7AS, 4AL (translocated from the original locus on 7BS), and 7DS (Yamamori *et al.*, 1994). The variants of Waxy (Wx-A1, Wx-B1, and Wx-D1) have been used to determine the effect of deficiencies on amylase content and starch pasting properties (Yamamori, 2009). However, the frequencies of variation at the Waxy locus are relatively low in modern wheat cultivars. In our system *TaWaxy-4A*, *TaWaxy-7A*, and *TaWaxy-7D* were successfully edited with high efficiency by CRISPR/SpCas9, and further research is planned to obtain homozygous strains and to test the starch with a view to enhancing the flour quality.

## Supplementary data

Supplementary data are available at *JXB* online.

Fig. S1. Schematic map of the vector pWMB110-SpCas9/LbCpf1/xCas9.

Fig. S2. Detection of mutations in *GUS*, *TaMTL-179*, *TaMTL-471*, *TaWaxy-296*, and *TaWaxy-830*.

Fig. S3. InDel mutations of *TaMTL* at the *TaMTL-179* site from edited T_0_ transgenic plants.

Fig. S4. InDel mutations of *TaMTL* at the *TaMTL-471* site from edited T_0_ transgenic plants.

Fig. S5. Detection of large fragment deletions from different *TaMTL* and *TaWaxy* homologous genes.

Fig. S6. Sequences of *TaMTL* at loci on the 4A chromosome in the wild-type and the edited plant QD33-3.

Fig. S7. Sequences of *TaMTL* at loci on the 4D chromosome in the wild-type and the edited plant QD33-3.

Fig. S8. InDel mutations of *TaWaxy* at the *TaWaxy-296* site from edited T_0_ transgenic plants.

Fig. S9. InDel mutations of *TaWaxy* at the *TaWaxy-830* site from edited T_0_ transgenic plants.

Fig. S10. Sequences of *TaWaxy* at loci on the 7A chromosome loci in the wild-type and the edited plant Xd350-15.

Table S1. Summary of the target sequences and mutations of *GUS* in T_0_ wheat plants obtained using the CRISPR/xCas9 editing system.

Table S2. PCR primers used for vector construction and editing identification.

Table S3. Frequency distribution of the mutations for *GUS* in T_0_ wheat plants using the CRISPR/SpCas9-D system.

Table S4. Off-target detection using designed sgRNAs for *GUS*, *TaMTL*, and *TaWaxy*.

erz529_suppl_Supplementary_Data
