# Supplementary material for: Efficient induction of haploid plants in wheat by editing of TaMTL using an optimized Agrobacterium-mediated CRISPR system
Source: J Exp Bot. 2019 Nov 24;71(4):1337–49. doi: 10.1093/jxb/erz529 (PMC7031065; doi:10.1093/jxb/erz529)
Supplement: erz529_suppl_Supplementary_Data [file erz529_suppl_supplementary_data.pdf]

Editing *TaMTL* gene induces haploid plants efficiently by optimized *Agrobacterium*-mediated CRISPR system in wheat

Huiyun Liu,[1, 2] Ke Wang,[1, *] Zimiao Jia,[1] Qiang Gong,[1] Zhishan Lin,[1] Lipu Du,[1] Xinwu Pei,[2] and Xingguo Ye,[1, *]
[1]Institute of Crop Science, Chinese Academy of Agricultural Sciences, Beijing 100081, China
[2]Biotechnology Research Institute, Chinese Academy of Agricultural Sciences, Beijing 100081, China
*Corresponding authors (wangke03@caas.cn, yexingguo@caas.cn)

Supplementary data:

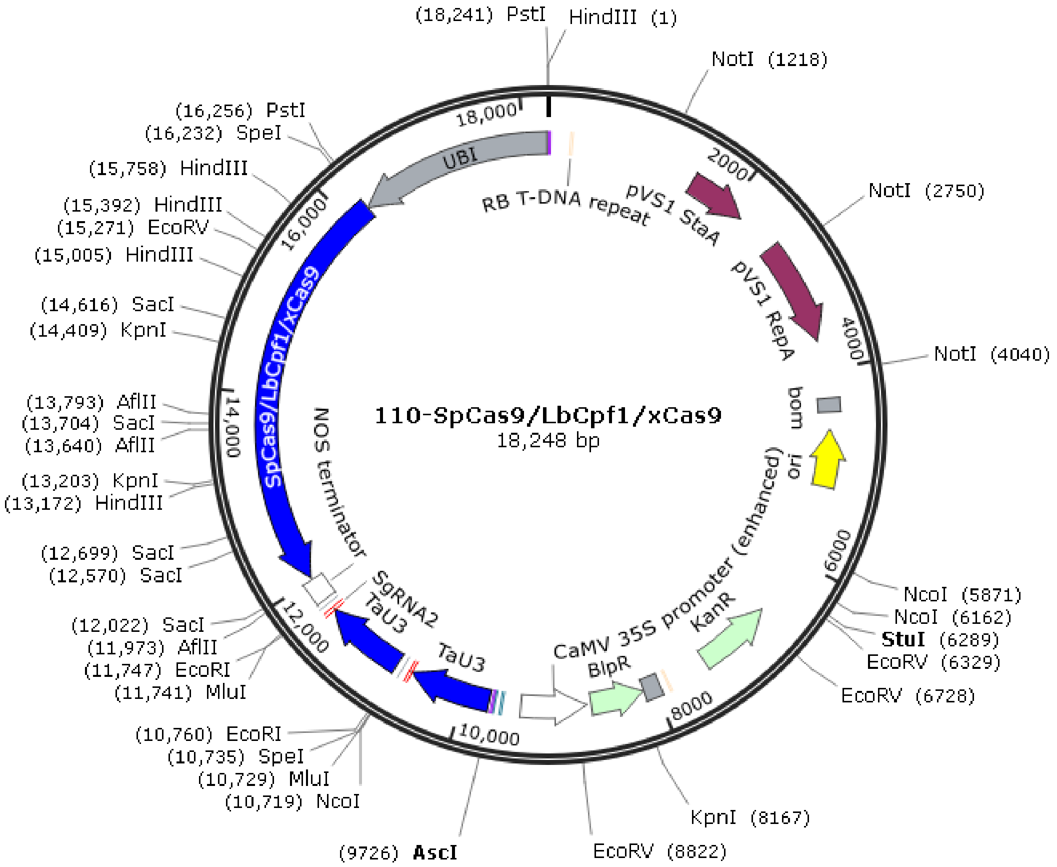

Figure S1 Schematic map of vector pWMB110-SpCas9/LbCpf1/xCas9. The vector is comprised of a SpCas9/LbCpf1/xCas9 expression cassette and the *phosphinothricin* (*bar*) gene, which was used for screening the transgenic wheat plants.

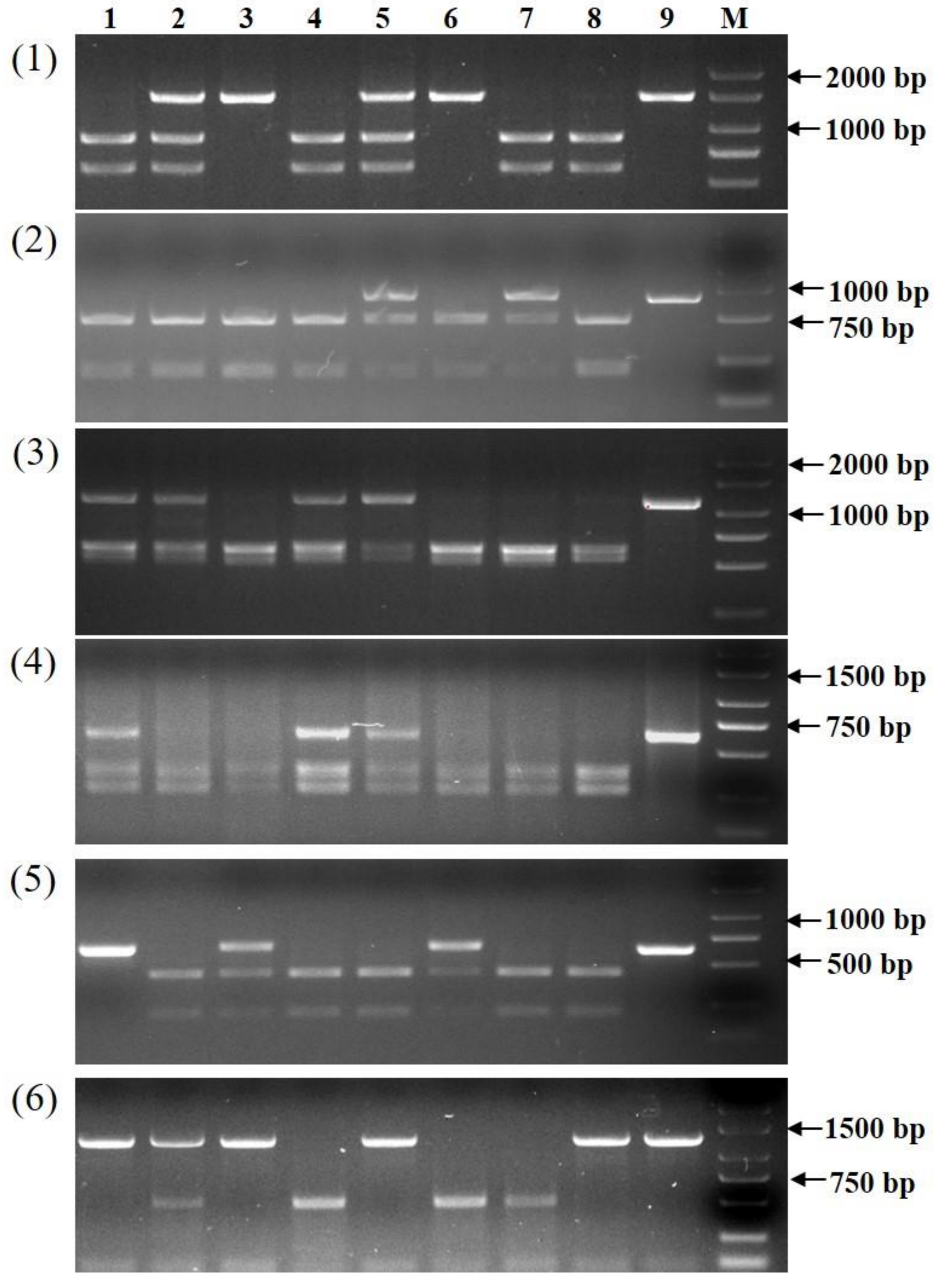

Figure S2 Detection of mutations in the *GUS* ((1) and (6)), *TaMTL-179* (2), *TaMTL-471* (3), *TaWaxy-296* (4) and *TaWaxy-830* (5) genes. Lanes (1)1–(5)7: digested DNA of PCR products amplified from different transgenic plants; lanes (1)3, (1)6, and (5)1: biallelic mutants; lanes (1)2, (1)5, (2)5, (2)7, (3)1, (3)2, (3)4, (3)5, (4)1, (4)4, (4)5, 5(3) and 5(6): heterozygous mutants; lanes (1)1, (1)4, (1)7, (2)1, (2)2, (2)3, (2)4, (2)5, (2)6, (3)3, (3)6, (3)7, (4)2, (4)3, (4)6, (4)7, 5(2), 5(4), 5(5) and 5(7): non-mutant plants; lanes (1)8–(5)8: digested PCR products from marker-free line H29 containing *GUS* and wild-type plants Fielder and Ningchun4; lanes (1)9–(5)9: undigested PCR products from wheat line H29 and wild-type plants Fielder and Ningchun4; lanes (6)1–(6)8: PCR products of *GUS* from the transgenic plants; lanes (6)2, (6)4, (6)6, and (6)7: plants with a large fragment deletion; lane (6)9: PCR product of *GUS* from the wheat line H29; lane M: 2-kb marker ladder.

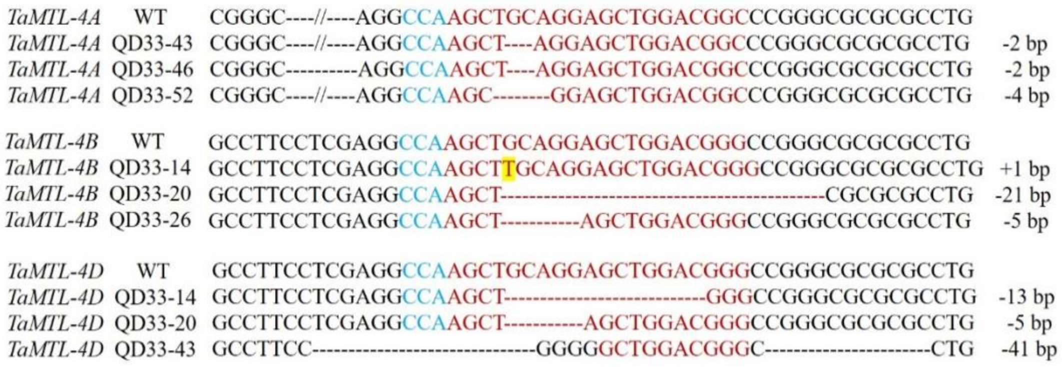

Figure S3 InDel mutations of the *TaMTL* gene at the *TaMTL-179* site from edited T_0 transgenic plants. WT: Fielder; QD33-14, QD33-20, QD33-26, QD33-43, QD33-46, and QD33-52: the *TaMTL*-edited plants.

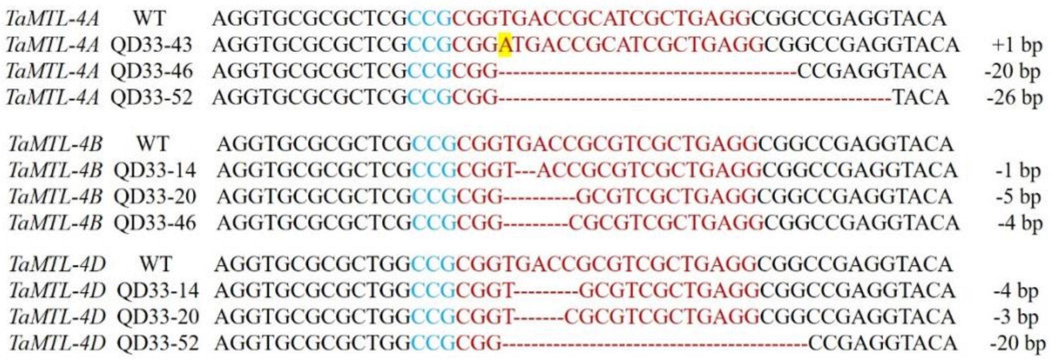

Figure S4 InDel mutations of the *TaMTL* gene at the *TaMTL-471* site from edited T_0 transgenic plants. WT: Fielder; QD33-14, QD33-20, QD33-43, QD33-46, and QD33-52: the *TaMTL*-edited plants.

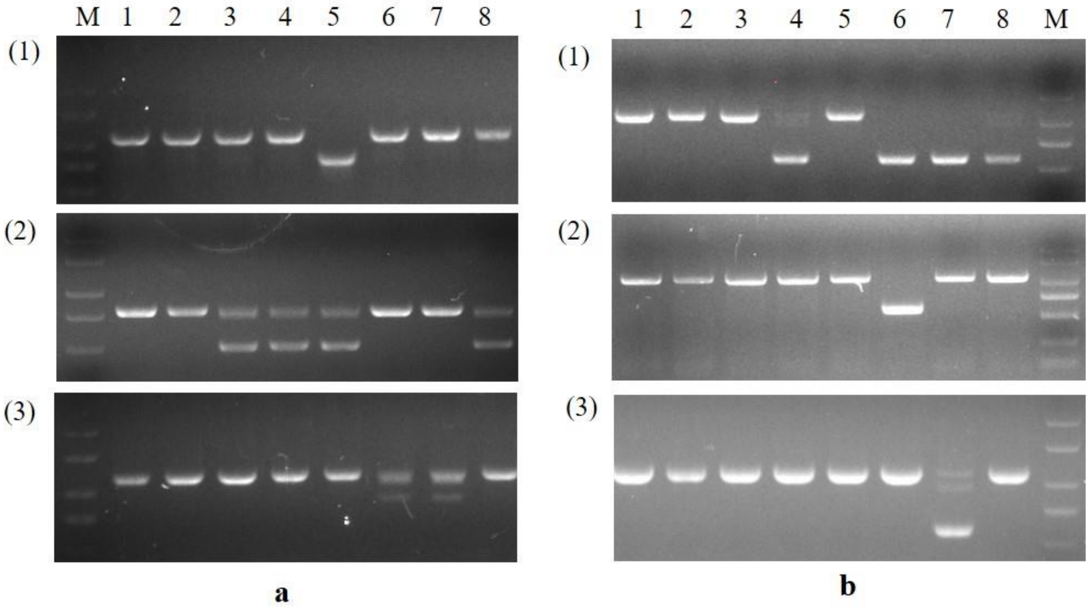

Figure S5 Detection of large fragment deletions from different *TaMTL* and *TaWaxy* homologous genes. (a) PCR products of the *TaMTL-4A* (1), *TaMTL-4B* (2) and *TaMTL-4D* (3) genes in transgenics (lanes 2–8) and *TaMTL* gene in wild-type Fielder (lane 1). Plants with a large fragment deletion in (1)5: *TaMTL-4A*; (2)3, (2)4 and (2)5: *TaMTL-4B*; and (3)6 and (3)7: *TaMTL-4D*. (b) PCR products of the *TaWaxy-4A* (1), *TaWaxy-7A* (2), and *TaWaxy-7D* (3) genes in transgenics (lanes 2–8) and *TaWaxy* gene in wild-type Ningchun4 (lane 1). Plants with a large fragment deletion in (1)4, (1)6, (1)7 and (1)8: *TaWaxy-4A*; (2)6: *TaWaxy-7A*; and (3)7: *TaWaxy-7D*. Lane M: 2-kb marker ladder.

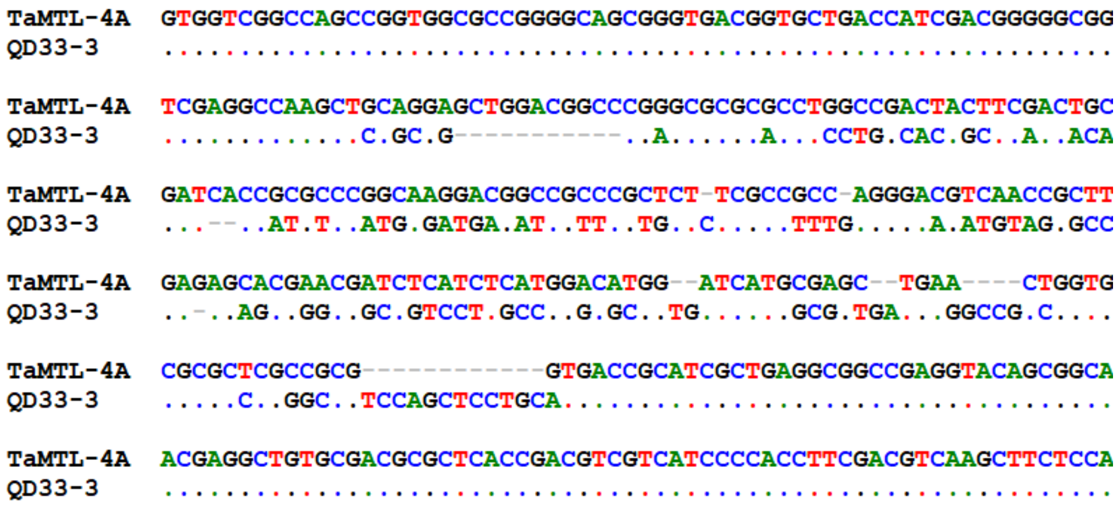

Figure S6 Sequence information of *TaMTL* gene at 4A chromosomal loci in the wild-type and the edited plant QD33-3.

```
TaMTL-4D   CCAGCCGGTGGCGCCGGGGCAGCGGGTGACGGTGCTGACCATCGACGGGGGCGGCATCCGCG
QD33-3     ............................................................

TaMTL-4D   AAGCTGCAGGAGCTGGACGGGCCGGGCGCGCCTGGCCGACTACTTCGACTGCATCGCCGG
QD33-3     .....A.C.CG..CA.------..C..A.-.T......--..A...C--.A....ATA.ATA

TaMTL-4D   CGCCCGGCAAGGACGGCTGCCCGCTCTTCGCCGCCAGGGACGTCAACCGCTTCTACCTCGAC
QD33-3     .ATGA.ATG..AT..TTC..G.T...AC.TTT..GG.AAGATGT.GGG..CAT.GT.GA.GT

TaMTL-4D   ACGATCTCATCTCATG-GACATGGATCGTGCGAGCTGAACTGGTGAT--TGATGTATGTATT
QD33-3     C..TC..TGC.GGGC.C.GTGATC..G.C.GTGATGAGG.C.CC.G.GC..G..CCG.CGA.

TaMTL-4D   T-----------GACCGCGTCGCTGAGGCGGCCGAGGTACAACGGCAAGTACCTGCACGGGA
QD33-3     CCAGCTCCTGCA..................................................

TaMTL-4D   GCGCTCACCGACGTGGTCATCCCCACCTTCGACGTCAAGCTTCTCCAGCCCATCATCTTCTC
QD33-3     ............................................................
```

Figure S7 Sequence information of *TaMTL* gene at 4D chromosomal loci in the wild-type and the edited plant QD33-3.

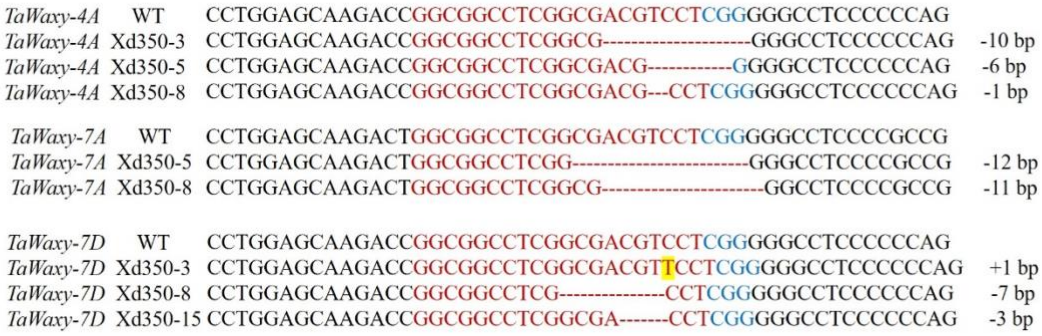

Figure S8 InDel mutations of the *TaWaxy* gene at the *TaWaxy-296* site from edited T_0 transgenic plants. WT: Ningchun4; Xd350-3, Xd350-5, Xd350-8, and Xd350-15: the *TaWaxy*-edited plants.

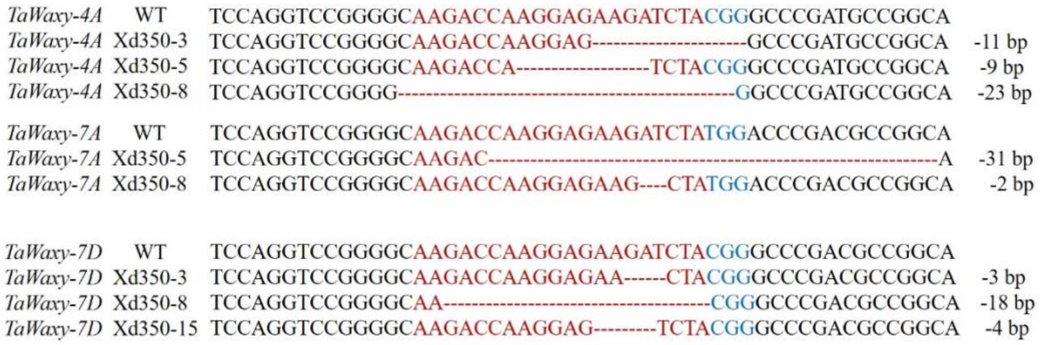

Figure S9 InDel mutations of the *TaWaxy* gene at the *TaWaxy-830* site from edited T0 transgenic plants. WT: Ningchun4; Xd350-3, Xd350-5, Xd350-8, and Xd350-15: the *TaWaxy*-edited plants.

```
TaWaxy-7A   ACCGGCGGTGCCTCTCCATGGTGGTGCGCGCCACGGGCAGCGGCGGCATGAACCTCGTGTT
Xd350-15    ............................................................

TaWaxy-7A   CCTCGGCGACGTCCTCGGGGGCCTCCCCGCCGCCATGGCCGTAAGCTTGCGCCACTGCCTT
Xd350-15    ............T--..TCTTGC...G.A.CTGGAAATT....C.AGCA.A.G.AAAGAC

TaWaxy-7A   AACGGGTGCCGTGTCCGTGCAGGCCAACGGTCACCGGGTCATGGTCATCTCCCCGCGCTAC
Xd350-15    G...A.GAG.TA.CTT.-ATC.AT.G....CG.G..ATCGG.CAC.T.....AG.AAGC..

TaWaxy-7A   CGAGGTATATATCCGCCACATGAATTATCACAATTCACATGCTCCTGCACATTTCTGCAAG
Xd350-15    .AGT.G.AG..C.TCA.C.TCTCG.ACCTGTC.ACG..C.TGAT....GAGA.C.A..C..

TaWaxy-7A   GACAGGTACGAGAGGGTGAGGTACTTCCACTGCTACAAGCGCGGGGTGGACCGCGTGTTCG
Xd350-15    TGTGATA.TTCAT.T.GCG.A..TA.A.CTC.GAGATGA...T..-..TC..AG.C..C.T

TaWaxy-7A   CGCCGTCGATCGAT-CAAGCTAGCTCCTCGTCGTCTCAACCCGCATGGTGTTTGATAATTT
Xd350-15    GA....T.GC.TGCA.GGA.AC.GCA.C...T..AA.GG.AG......CTGCA.GA.GAAA

TaWaxy-7A   CCGGGGCAAGACCAAGGAGAAGATCTATGGACCCGACGCCGGCACCGACTACGAGGACAAC
Xd350-15    .G.C..GG..G..CCC...--........................................

TaWaxy-7A   GTGCCCAGGATCCTCGACCTCAACAACAACCC
Xd350-15    ................................
```

Figure S10 Sequence information of *TaWaxy* gene at 7A chromosomal loci in the wild-type and the edited plant Xd350-15.

Table S1 Summary of the target sequences and mutation of the *GUS* gene in wheat T_0 plants obtained using the CRISPR/xCas9 editing system.

Target loci	Promoter	PAM-guide sequence (5'-3')	Transgenic plants	Mutant plants	Mutation rate %	Genotype
**xCas9 3.7**						
GUS-1718	*TaU3*	CCGCCAGGAATTTCACGCGT**GGA**	71	0	0	71WT
GUS-1159	*TaU3*	ACACTCCTATCAACGGCCGT**GGC**	67	0	0	67WT
GUS-1719	*TaU3*	CGCCAGGAATTTCACGCGTG**GAA**	79	0	0	79WT
GUS-1156	*TaU3*	TCGTTAAAGCCACGGCCGTT**GAT**	79	0	0	79WT

Table S2 PCR primers used for vector construction and editing identification in this study.

Primer name	Primer sequence (5′→3′)	Product size (bp)	Application
Cas9F	TCAAGGCTCTTGTTCGTCAGCA	1026	Detecting CRISPR/Cas9 or CRISPR/xCas9 construct
Cas9R	TTGCCGCTCTGCTTATCCCTGA		
Cpf1F	ATCTCCCTCTTCAGAAAGA	802	Detecting CRISPR/Cpf1 construct
Cpf1R	AGATATCTTTACTTATCGTT		
TaU3F	GAATTCATCCTCACGTTCAACACC	857	Amplification the TaU3 promoter
TaU3R	CCTGTTCAAGTAAGGTCGTGCTTC		
TaU6F	GACCAAGCCCGTTATTCTGACAG	844	Amplification the TaU6 promoter
TaU6R	CGTGGCTCCATGTAGGACTTTC		
OsU6aF	CCAAGTGAACAGTATTCCTATA	816	Amplification the OsU6a promoter
OsU6aR	TTCCACACAACATACGAGCCG		
F1	CGTTCATTCGTTCTAGATCGGAG	1320	PCR/RE detection for *GUS-788* (Eco105 I), *GUS-789* (Eco105 I), and *GUS-118* (Dra I)
R1	ATCCACGACCGACACTTTCACG		
F2	CAAGGAAATCCGCAACCATATC	996	PCR/RE detection for *GUS-1156* (Eco52 I), *GUS-1159* (Eco52 I),
R2	TCAAACGTCCGAATCTTCTCCC		
F3	GAACTCGACCCACAGAAGCGT	652	PCR/RE detection for *GUS-1718* (Mlu I),

R3	TGTATAATTGCGGGACTCTAATC		*GUS-1719* (Mlu I), and *GUS-2046* (Nhe I)
F4	CTAGCAAACCCACCAATTAC	553	PCR/RE detection for *TaMTL4A-179* (Pst I),
R4	GATCCAAGTATAAAATTAGCATAT		and *TaMTL4A-471* (Eco91 II)
F5	TTCAATCGATGGCAAGCTACTGGG	781	PCR/RE detection for *TaMTL4B-179* (Pst I),
R5	CGTCATCGCCACCATCGTCTGC		and *TaMTL4B-471* (Eco91 II)
F6	GTCAACCGAGGTGCCGTCAGTTTG	903	PCR/RE detection for *TaMTL4D-179* (Pst I),
R6	CGTCATCGCCACCATCGTCTGC		and *TaMTL4D-471* (Eco91 II)
F7	CCCCGAAGCAACAAAGCCGGAAAG	1167	PCR/RE detection for *TaWaxy4A-296* (Zra I),
R7	TGCAGAACGCTACCTGGACATG		and *TaWaxy4A-830* (Bgl II)
F8	GAAACCGCACCGATTCGACCGGCG	760	PCR/RE detection for *TaWaxy7A-296* (Zra I),
R8	AGTTGTTCTTGATCTTACCGTAGG		and *TaWaxy7A-830* (Bgl II)
F9	GTGCCTCTCCATGGTGGTGCGCG	1041	PCR/RE detection for *TaWaxy7D-296* (Zra I),
R9	TGAACCGCAAAATTGATATGCCTG		and *TaWaxy7D-830* (Bgl II)
TaADPF	GCTCTCCAACAACATTGCCAAC	201	Quantification of endogenous gene *TaADP*
TaADPR	GCTTCTGCCTGTCACATACGC		
*MTL*4AF	TCATCACCGCCATGATCACCGCG	161	Quantification of *MTL4*A RNA
*MTL*4AR	ATCTTGCCGTGCAGGTACTTGCC		

*MTL*4BF	ACGTCAACCGCTTCTACCTCGACA	129	Quantification of *MTL4*B RNA
*MTL*4BR	CTCGCCGAGCATGCTCCTGATCTT		
*MTL*4DF	CCCATCATCTTCTCCACATACGAC	134	Quantification of *MTL4*D RNA
*MTL*4DR	ATGAGGTTGTACTCGCGCTCCTTG		

Table S3 Off-target detection using designed sgRNA for *GUS, TaMTL,* and *TaWaxy* genes.

Target gene	Genomic location	Off-target region*	Primers sequence (5' to 3')	Mutations
GUS-Off-target1	Chromosome 1B: 667,829,586-667,829,602	ACGTACGTCGAGGACACACACGG	F: 5'-TGATTAGCACTAATCAATGT-3'<br>R: 5'-TAGTAGTAGTAAATGGAGT-3'	No
GUS-Off-target2	Chromosome 4A: 420,031,185-420,031,205	TTTGCTACAGCTTTGATCTTTCTTTAA	F: 5'-TGCTGGAATAGTGAAGC-3'<br>R: 5'-AATTTAAGGGTAAGAGA-3'	No
GUS-Off-target3	Chromosome 7B: 673,667,427-673,667,447	TTTGCTACAGCTTTGATCTTTCTTTAA	F: 5'-ATACAGAAGCCAGCAGAT-3'<br>R: 5'-AATTTAAGGGTAAGAGAT-3'	No
GUS-Off-target4	Chromosome 3B: 390,919,793-390,919,813	TTTGCTAAAGCTTTGATCTTTCTTTAA	F: 5'-TTGCCGCCCTGCCATGGGA-3'<br>R: 5'-CTTGCCTGCATAGTGGGT-3'	No
GUS-Off-target5	Chromosome 1B: 628,855,584-628,855,601	CCTCCTATCAACGGCCGTCCGGC	F: 5'-AGCTCTCTGTTAGTGCGTG-3'<br>R: 5'-CGTTGCACGCGTGGTTTGT-3'	No
GUS-Off-target6	Chromosome 4B: 385,935,844-385,935,860	ATCTTAAAGCCACGGCCGTCTCT	F: 5'-TAAGAGCAAAAGAAGGAT-3'<br>R: 5'-TGTGCGGCGAGTGGAATG-3'	No
TaMTL-Off-target1	TraesCS4B02G011600	CCTCGTCCAGCTCCTGCAGCTCC	F:5'-GGGGATAGAAAAGAAATCAT-3'<br>R:5'-CGCCTGTCCTCGTCCACTCC-3'	No
TaMTL-Off-target2	TraesCS4A02G302200	CCTCGTCCAGCTCCTGCAGCTGC	F: 5'-AGAAAAGGAAGGGCTAATC-3'<br>R: 5'-TCTGTGAAGTCGCTGTCAA-3'	No
TaMTL-Off-target3	TraesCS4D02G009700	CCCTCGTCCAGCTCCTGCAGCTG	F: 5'-GGATATATAGAAAAGGAAG-3'<br>R: 5'-GCCAGTGGAGTAAATGAG-3'	No
TaMTL-Off-target4	TraesCS1B02G061400	CCTTCGTCCAGCTCCTGCAGCTG	F: 5'-TAGAAAAGGAAGGGCTAA-3'<br>R: 5'-CTCCTGTCCTCGTCCACT-3'	No
TaMTL-Off-target5	TraesCS6B02G346800	CACGTCCAGCTCCTGCAGCAGGG	F: 5'-AGCGGGGTGGCGCGGATCT-3'<br>R: 5'-GCGAGCACACGCCAACCT-3'	No
TaMTL-Off-target6	Chromosome 6D:	TCATCGCGATGCGGTCACCGCGG	F: 5'-TCGCCTGCAGCTGCCAT-3'	No

	433,758,923-433,758,941		R: 5’-TCGCAGCACCGCCAATGAT-3’	
TaWaxy-Off-target1	TraesCS2D02G311500<br>TraesCS2A02G313000	GACGGCCTCGGCGACGTCCCAGG	F: 5’-CCCCGTTGCAACGCAAACA-3’<br>R: 5’-TGGAGCGCAACCCGGCC-3’	No
TaWaxy-Off-target2	Chromosome 5D:<br>197,024,951-197,024,968	CAGGGCCTCGGCGACGTCCTGGG	F: 5’-TCCTAGTGAACTCCCTAAC-3’<br>R: 5’-CCACTTTAAGTTTGACGGT-3’	No
TaWaxy-Off-target3	Chromosome 3D:<br>123,964,171-123,964,188	CCGGGCGGCCTCGGCGACGTGGC	F: 5’-GTACTGTTGATGAAGATGA-3’<br>R: 5’-AACCTTTTTGCCGAACTGG-3’	No
TaWaxy-Off-target4	Chromosome 2D:<br>307,826,867-307,826,884	CGGTGGCGGCCTCGGCGACGTGG	F: 5’-ACGGCGGGGCGCGCGTT-3’<br>R: 5’-GATTGGATCGGGGGTAT-3’	No

*The positions mismatching with the preselected targets are shaded in yellow, and the PAM motifs are shaded in green.
